# Supplementary material for: The causal role of circulating inflammatory markers in osteoporosis: a bidirectional Mendelian randomized study
Source: Front Immunol. 2024 Jul 18;15:1412298. doi: 10.3389/fimmu.2024.1412298 (PMC11291241; doi:10.3389/fimmu.2024.1412298)
Supplement: Supplementary file 1 [file DataSheet_1.zip › supplementary material/OSTEOPOROSIS-CIM/GCST90274769.tsv.gz_forest.pdf]

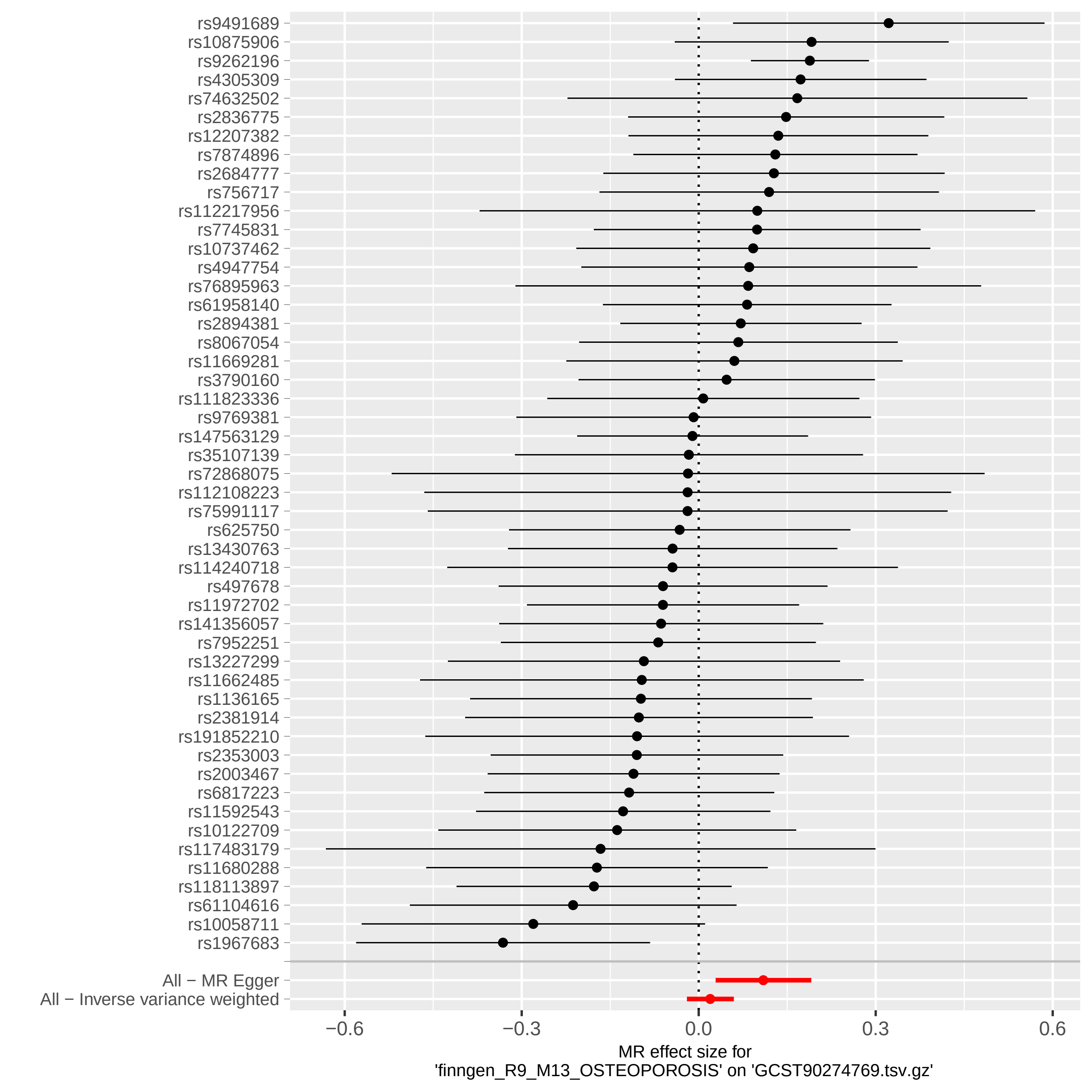
rs9491689
rs10875906
rs9262196
rs4305309
rs74632502
rs2836775
rs12207382
rs7874896
rs2684777
rs756717
rs112217956
rs7745831
rs10737462
rs4947754
rs76895963
rs61958140
rs2894381
rs8067054
rs11669281
rs3790160
rs111823336
rs9769381
rs147563129
rs35107139
rs72868075
rs112108223
rs75991117
rs625750
rs13430763
rs114240718
rs497678
rs11972702
rs141356057
rs7952251
rs13227299
rs11662485
rs1136165
rs2381914
rs191852210
rs2353003
rs2003467
rs6817223
rs11592543
rs10122709
rs117483179
rs11680288
rs118113897
rs61104616
rs10058711
rs1967683
All − MR Egger
All − Inverse variance weighted
−0.6
−0.3
0.0
0.3
0.6
MR effect size for
'finngen_R9_M13_OSTEOPOROSIS' on 'GCST90274769.tsv.gz'
